# Changes in sleep quality of children with epilepsy and anxiety of their caregivers after COVID-19 infection: a case-series report

**DOI:** 10.3389/fped.2023.1239322

**Published:** 2023-08-22

**Authors:** Dan Li, Yongjing Shi, Bo Wang, Jing Zhou, Xueying Wang, Shaoping Huang, Lin Yang

**Affiliations:** ^1^ Department of Pediatrics, The Second Affiliated Hospital of Xi’an Jiaotong University, Xi’an, China; ^2^ Department of Pediatrics, Xi’an Gaoxin Hospital, Xi’an, China

**Keywords:** COVID-19, epilepsy, anxiety, sleep habits, questionnaire survey

## Abstract

**Objective:**

To study the changes in epileptic seizures and sleep quality in children with epilepsy (CWE) and the changes in anxiety of their caregivers after infection with COVID-19.

**Methods:**

Outpatients and inpatients of CWEs were selected as subjects and a questionnaire survey was used to carry out this case-series study. The demographic information of the CWEs and their caregivers, information about epilepsy, and information about the vaccination, infection, and treatment of COVID-19 were collected. The changes in sleep quality of CWEs and the changes in anxiety of their caregivers were assessed by the Child Sleep Habits Questionnaire (CSHQ) and Caregiver Anxiety Scale (CAS). Risk factors affecting sleep habits in CWEs and caregiver anxiety were further analyzed by one-way analysis of variance.

**Results:**

A total of 312 children were included in the study. Among them, 134 patients (42.9%) were female. The average age of the children was 9.30 ± 3.88 years, and the duration of epilepsy was 4.59 ± 3.36 years. A total of 221 of the 312 children were infected with COVID-19, and all the infected children developed fever, which lasted for 1.71 ± 1.13 days. 10 children were satisfied with controlled seizures for more than 1 year and relapsed after COVID-19 infection (4.2%), 4 cases (3.6%) with increased seizures, and 8 children with reduced seizures (7.7%), 17 children (7.7%) had no change in seizures, and 182 children (82.3%) remained seizure-free after the COVID-19 infection. The average sleep time of the CWEs was 9.25 ± 1.04 h and the average total score of the CSHQ was 37.25 ± 5.19, among which 44 cases (14.1%) had more than 41 points. As the result of the CAS, 16 of them (5.13%) scored above 50 and the average total score was 31.49 ± 8.09. The control of seizures, age of onset, types of anti-seizure medicines (ASMs), and seizure duration were risk factors affecting sleep quality. Accordingly, the score of CAS was significantly lower when there was more than one caregiver who cared for the CWE.

**Conclusions:**

COVID-19 infection did not cause an increase in seizures in CWEs, nor did it worsen their sleep quality of them or aggravate the anxiety of their caregivers.

## Introduction

Epileptic seizures refer to clinical manifestations caused by an abnormal, excessive, or synchronous discharge of neurons mainly located in the cerebral cortex. According to reports, more than 50 million people worldwide suffer from epilepsy, and the prevalence of this disease is 0.7%–1.0% (1). In children, the incidence of epilepsy is highest in the first year of life but decreases to adult levels by the age of 10 years (2). Epilepsy and sleep have a close and complex relationship (3), sleep deprivation is a common predisposing factor for epilepsy recurrence (4), and sleep problems are common in infancy, childhood, and adolescence (5). Caring for children with epilepsy is not easy, and studies have pointed out that the caregivers of children with epilepsy (CWE) face many obstacles and challenges, and experience severe stress, fear, and anxiety (6). These negative emotions are not only detrimental to the physical and mental health of the caregivers themselves but also detrimental to caring for CWEs, increasing the risk of epilepsy recurrence, worsening the level of seizures, and increasing the risk of psychological comorbidities in CWEs.

COVID-19 is prevalent around the world. Studies have shown that measures to respond to the pandemic COVID-19 may lead to lock-down, inconvenient transportation, unavailable of medical treatment, inaccessibility of anti-seizure medicines (ASM), and reduced income. These factors would in turn to worse sleep quality and greater anxiety (7, 8). However, it has not been reported whether the COVID-19 infection itself can cause changes in the sleep quality of CWEs and the anxiety of their caregivers. This study used a questionnaire survey to carry out a case series study to illustrate the changes in seizures and sleep quality of CWEs, as well as the changes in the anxiety of their caregivers after infection with COVID-19.

## Methods and materials

### Study design and patients

From January 10, 2023, to March 3, 2023, outpatients and inpatients of CWEs and their caregivers in the Pediatric Epilepsy Department of the Second Affiliated Hospital of Xi’an Jiaotong University were taken as the research subjects and a case-series study was carried out using the questionnaire survey method. Inclusion criteria: (1) diagnosed with epilepsy, and the course of the disease is more than 1 year; (2) age was between 4 and 12 years old; (3) caregivers were mentally and cognitively normal, and can accurately fill in the form; (4) infected with COVID-19 (diagnostic criteria: positive test for COVID-19 nucleic acid or antigen, definitive contact history, and corresponding clinical manifestations and signs). Exclusion criteria: (1) with severe abnormalities in heart, liver, and kidney function; (2) with convulsions or disturbance of consciousness within 1 month; (3) the contents of the questionnaire cannot be filled in completely.

The study was approved by the Ethics Committee of the Second Affiliated Hospital of Xi’an Jiaotong University (Ethics Committee Number: 2023244, Date: January 3, 2023), written informed consents were obtained from the enrolled patients and their parents, and the research implementation complied with the Declaration of Helsinki.

### Questionnaire survey

To make sure patients clearly understand the content of the questionnaire and to ensure the quality of the questionnaire survey, the questionnaire was completed face-to-face on-site. The questionnaire survey was administered by physicians with more than 3 years of professional experience in pediatric neurology after receiving training about conducting questionnaire research.

The questionnaire used consisted of four parts, which are: (1) The basic information of the CWEs and their caregivers, including demographic information, epilepsy status (age of onset, type of seizure, longest seizure time ever, seizure frequency, epilepsy syndrome, comorbidities, cranial magnetic resonance, anti-seizure medicines used, etc.); (2) Information related to COVID-19 infection (duration of fever, the basis for diagnosing COVID-19, whether imaging examinations have involved lungs, etc.), and epileptic seizures within 1 month after COVID-19 infection; (3) questionnaire on sleep habits of children with epilepsy; (4) anxiety self-rating scale for caregivers of CWEs.

The Children's Sleep Habits Questionnaire (CSHQ) was used, which is a retrospective caregiver report questionnaire developed by Owens et al. (9). The CSHQ mainly investigates caregivers’ assessment of children's sleep behaviors in the past week. The questionnaire has a total of 8 dimensions, which are divided into sleep resistance, sleep onset delay, sleep duration, sleep anxiety, sleep arousal, sleep-associated disorders, sleep-disordered breathing, and daytime sleepiness. There are 33 items in total, and each item is scored on a 3-point scale. Except for some items, generally, 3 points were scored for “always” (5–7 times a week), 2 points were scored for “sometimes” (2–4 times a week), and 1 point was scored for “rarely” (0–1 time per week), and the higher the score, the more sleep problems. The total score is the sum of 33 items, with a CSHQ score >41 as the cut-off value, and a score higher than 41 was considered to have sleep habit problems.

The Caregiver Anxiety Scale (CAS) was used for the anxiety self-rating scale for caregivers of CWEs. The scale has a total of 20 items. All items are divided into four grades: none, occasional, sometimes, and always. Correspondingly, 4 points were used, and the total score was the sum of the scores of each item of the 20 items. Finally, the total score was multiplied by 1.25 and rounded to an integer to obtain the standard score. A standard score <50 was considered normal; a standard score of 50–60 was taken as mild anxiety; 61–70 was moderate anxiety; and more than 70 was severe anxiety.

### Statistical analysis

SPSS (version 29.0) was used for Statistical Analysis. Measurement data were reported as mean ± standard deviation (M ± SD), and count data were reported as rate, *n* (%). Finally, patients were grouped according to whether they were infected with COVID-19, age of onset, longest duration of epileptic seizures, types of ASMs, duration of epilepsy control, and number of caregivers. One-way analysis of variance was used for comparison between groups, and the multiple comparisons was carried out by the LSD method. A *p*-value <0.05 was considered statistically significant.

## Results

A total of 350 questionnaires were distributed, 335 questionnaires were returned, and finally, a total of 312 questionnaires met the inclusion and exclusion criteria and were included in the study.

Table 1 reported the first part of the questionnaire, the basic information about the CWEs and their caregivers, and Table 2 reported the subjects’ COVID-19 vaccination status, COVID-19 infection and treatment status, and post-infection conditions of the seizures. Table 3 reported the results of the CSHQ and the CAS.

**Table 1:** Demographic data and basic information about epilepsy.

Items	
Male/Female	178/134
Age, mean ± standard deviation, (year)	9.30 ± 3.88
Education level, *n* (%)	
Drop out	21 (6.7%)
Kindergarten	64 (20.5%)
Primary school	227 (72.8%)
Caregiver, *n* (%)	
Mother/Father	34 (10.9%)
Parents	250 (80.1%)
Grandmother/grandfather	16 (5.1%)
Grandparents	12 (3.8%)
Course of epilepsy, mean ± standard deviation, (year)	4.59 ± 3.36
The longest seizure duration was more than 5 min, *n* (%)	
No	252 (80.8%)
Yes	60 (19.2%)
Classification of seizures, *n* (%)	
Generalized epilepsy	177 (56.7%)
Focal epilepsy	130 (41.7%)
Unknown	5 (1.6%)
Epilepsy syndrome, *n* (%)	
Sel-ECT	102 (32.7%)
Lennox-gastaut syndrome	15 (4.8%)
CAE	23 (7.4%)
JME	17 (5.5%)
Dravet syndrome	18 (5.8%)
Doose syndrome	11 (3.5%)
Genetic generalized epilepsy with generalized tonic-clonic seizures	45 (14.4%)
Unknown	81 (26.0%)
Control of seizures, *n* (%)	
Completely controlled ≥ 3 years	95 (30.4%)
3 years> completely controlled ≥1 year	77 (24.7%)
Completely controlled <1 year	71 (22.8%)
Uncontrolled	69 (22.1%)
More than three types of ASMs, *n* (%)	
Yes	242 (77.6%)
No	70 (22.4%)
Results of MRI, *n* (%)	
No obvious abnormalities were found	260 (83.3%)
With abnormalities	38 (12.2%)
With no results	14 (4.5%)
Absence of comorbidities, *n* (%)	267 (85.6%)
Presence of comorbidities, *n* (%)	45 (14.4%)
Cerebral palsy	8 (17.8%)
Mental retardation	33 (73.3%)
ADHD	4 (8.9%)
Family history of epilepsy, *n* (%)	
Yes	10 (3.2%)
No	302 (96.8%)

Sel-ECT, self-limited epilepsy with centrotemporal spikes; CAE, childhood absence epilepsy; JAE, juvenile absence epilepsy; JME, juvenile myoclonic epilepsy; GGE, genetic generalized epilepsy; ASMs, anti-seizure medicines; ADHD, attention-deficit hyperactivity disorder.

**Table 2:** COVID-19 vaccination, infection, and treatment.

Items	
COVID-19 infection, *n* (%)	
Yes	221 (70.8%)
No	91 (29.2%)
Duration of fever, mean ± standard deviation, (day)	1.7 ± 1.1
Basis for the diagnosis of COVID-19 infection	
Nucleic acid testing (*N*AT)	62 (28.1%)
Antigens detection	14 (6.3%)
Symptoms and epidemiology	145 (65.6%)
Seizures within 1 month of COVID-19 infection, *n* = 221	
The seizures recurred	10 (4.2%)
The number of seizures increased	4 (1.8%)
The number of seizures decreased	8 (3.6%)
The number of seizures unchanged	17 (7.7%)
Absence of seizures	182 (82.3%)

**Table 3:** The results of the children's sleep habits questionnaire and the caregiver anxiety scale, *n* = 312.

Items	
Average sleep duration, mean ± standard deviation, (minutes)	9.25 ± 1.04
Total score, mean ± standard deviation	37.25 ± 5.19
Score of each item, mean ± standard deviation	
Sleep resistance	8.26 ± 2.50
Sleep onset delay	1.16 ± 0.38
Sleep duration	3.45 ± 1.12
Sleep anxiety	4.96 ± 1.33
Sleep arousal	3.18 ± 0.60
Sleep-associated disorders-parasomnias	6.87 ± 1.04
Sleep disordered breathing	3.11 ± 0.42
Daytime sleepiness	8.46 ± 1.39
The caregiver anxiety scale	31.49 ± 8.09

The risk factors affecting the sleep quality of CWEs and their caregivers’ anxiety were shown in Table 4.

**Table 4:** Analysis of factors affecting CSHQ of CWEs and CAS of their caregivers.

		Average sleep duration (hours)	Total score	Sleep resistance (6)	Sleep onset delay (1)	Sleep duration (3)	Sleep anxiety (4)	Sleep arousal (3)	Sleep-associated disorders-parasomnias (6)	Sleep disordered breathing (3)	Daytime sleepiness (8)	CAS
Age of onset	Within 1 year	9.43 ± 1.22	38.76 ± 5.7	9.14 ± 2.61	1.11 ± 0.31	3.65 ± 1.51	5.11 ± 1.26	3.14 ± 0.42	7.05 ± 1.31	3.19 ± 0.70	8.59 ± 1.80	32.64 ± 8.66
	1–3 years	9.35 ± 0.97	36.86 ± 4.43	8.39 ± 2.52	1.12 ± 0.38	3.32 ± 0.83	5.00 ± 1.30	3.19 ± 0.63	6.69 ± 0.81	3.10 ± 0.30	8.33 ± 1.03	31.39 ± 7.61
	4–6 years	9.36 ± 1.02	37.64 ± 6.16	8.21 ± 2.57	1.15 ± 0.50	3.39 ± 1.10	5.11 ± 1.48	3.23 ± 0.66	7.01 ± 1.29	3.14 ± 0.54	8.62 ± 1.50	31.41 ± 8.60
	7–14 years	8.95 ± 1.04	36.79 ± 5.02	7.73 ± 2.30	1.10 ± 0.31	3.60 ± 1.28	4.70 ± 1.29	3.16 ± 0.59	6.93 ± 0.93	3.07 ± 0.25	8.46 ± 1.53	31.19 ± 8.14
	*F* value	3.493	1.624	2.949	0.26	1.502	1.58	0.235	2.227	0.848	0.748	0.293
	*p* value	0.016*	0.184	0.033*	0.854	0.214	0.194	0.872	0.085	0.469	0.524	0.83
The longest duration of an episode	≤5 min	9.22 ± 1.05	37.40 ± 5.34	8.30 ± 2.49	1.13 ± 0.40	3.48 ± 1.16	5.00 ± 1.33	3.18 ± 0.60	6.87 ± 1.04	3.11 ± 0.44	8.52 ± 1.45	30.01 ± 7.11
	>5 min	9.41 ± 1.00	36.58 ± 4.53	8.08 ± 2.57	1.08 ± 0.28	3.32 ± 0.95	4.78 ± 1.38	3.20 ± 0.61	6.90 ± 1.04	3.12 ± 0.32	8.25 ± 1.10	37.69 ± 9.00
	*F* value	1.686	1.21	0.354	0.753	1.075	1.22	0.061	0.055	0.008	1.775	50.64
	*p* value	0.272	0.27	0.552	0.386	0.301	0.27	0.805	0.815	0.927	0.184	<0.01
Types of using ASMs	≤2 types	9.24 ± 1.07	37.06 ± 5.13	8.09 ± 2.37	1.13 ± 0.39	3.46 ± 1.10	4.90 ± 1.32	3.16 ± 0.60	6.87 ± 1.03	3.12 ± 0.45	8.45 ± 1.39	31.37 ± 8.27
	>2 types	9.28 ± 0.96	37.93 ± 5.42	8.87 ± 2.87	1.09 ± 0.33	3.43 ± 1.20	5.16 ± 1.42	3.25 ± 0.61	6.88 ± 1.04	3.09 ± 0.29	8.53 ± 1.40	31.91 ± 7.42
	*F* value	0.049	1.488	5.248	0.671	0.044	2.071	1.088	0.009	0.281	0.187	0.24
	*p* value	0.824	0.223	0.023*	0.413	0.833	0.151	0.298	0.925	0.597	0.665	0.625
Duration of seizure control	≥3 years	9.21 ± 1.04	36.35 ± 4.67*	7.94 ± 2.42	1.09 ± 0.28	3.33 ± 0.93	4.84 ± 1.31	3.10 ± 0.50	6.65 ± 1.10	3.07 ± 0.26	8.37 ± 1.32	30.69 ± 7.46
	1–3 years	9.17 ± 1.03	37.26 ± 4.84	8.34 ± 2.60	1.17 ± 0.47	3.44 ± 1.01	5.00 ± 1.30	3.20 ± 0.51	6.90 ± 0.91	3.10 ± 0.30	8.45 ± 1.32	31.30 ± 7.84
	Within 1 year	9.27 ± 0.90	37.53 ± 6.15	8.09 ± 2.33	1.10 ± 0.39	3.48 ± 1.31	4.94 ± 1.43	3.18 ± 0.63	7.00 ± 1.05	3.12 ± 0.54	8.45 ± 1.51	30.67 ± 7.06
	Uncontrolled	9.42 ± 1.19	38.35 ± 5.22*	8.83 ± 2.64*	1.13 ± 0.38	3.63 ± 1.32	5.10 ± 1.36	3.30 ± 0.80*	7.10 ± 1.04*	3.19 ± 0.59	8.65 ± 1.47	33.87 ± 9.92*
	*F* value	0.763	2.025	1.759	0.728	0.963	0.506	1.469	2.709	1.103	0.534	2.422
	*p* value	0.515	0.11	0.155	0.536	0.411	0.679	0.223	0.045	0.348	0.659	0.066
COVID-19 infection	Yes	9.17 ± 1.08	36.99 ± 5.01	8.16 ± 2.57	1.12 ± 0.38	3.43 ± 1.08	4.89 ± 1.40	3.14 ± 0.52	6.88 ± 1.05	3.10 ± 0.38	8.48 ± 1.48	32.00 ± 8.61
	No	9.45 ± 0.92	37.87 ± 5.60	8.49 ± 2.34	1.12 ± 0.39	4.38 ± 1.22	5.11 ± 1.18	3.27 ± 0.76	6.85 ± 1.01	3.14 ± 0.51	8.42 ± 1.15	30.24 ± 6.53
	*F*	4.738	1.839	1.161	0.001	0.101	1.715	3.025	0.078	0.682	0.147	3.035
	*p* value	0.03*	0.176	0.282	0.978	0.751	0.191	0.083	0.78	0.41	0.701	0.082
Number of caregivers	1	9.21 ± 1.12	37.00 ± 4.47	8.16 ± 2.76	1.16 ± 0.45	3.49 ± 1.18	4.96 ± 1.36	3.18 ± 0.47	6.88 ± 1.02	3.09 ± 0.29	8.32 ± 0.97	34.93 ± 7.67
	≥2	9.27 ± 1.03	37.32 ± 5.36	8.29 ± 2.45	1.11 ± 0.36	3.44 ± 1.11	4.96 ± 1.36	3.19 ± 0.63	6.87 ± 1.04	3.12 ± 0.45	8.50 ± 1.47	30.74 ± 8.00
	*F* value	0.598	0.421	0.468	0.355	0.120	0.254	0.052	0.353	0.156	0.463	6.835
	*p* value	0.550	0.657	0.627	0.702	0.887	0.776	0.949	0.703	0.855	0.630	0.001

* *p *< 0.05, with a significant difference.

According to the identity of the caregiver, age of onset, type of oral ASMs, longest seizure duration, infection with COVID-19, duration of epilepsy control, and type of ASMs taken, the sleep quality and caregiver anxiety self-rating scale of children with epilepsy were assessed. Analysis of variance between groups was carried out, and confounding factors such as gender and age were excluded. The detailed results are shown in Table 4.

In the comparisons between the groups, it was found that the total score of sleep assessment in children with uncontrolled seizures was 38.35 ± 5.22, which was significantly higher than that in children with seizures controlled for more than 3 years, *p *= 0.017. The sleep time of children with onset age over 7 years old was 8.95 ± 1.04 h, which was shorter than that of other groups, *p *= 0.016. The Bedtime Resistance score of children with onset of epilepsy under 1-year-old was 9.14 ± 2.61, which was higher than that of other groups, *p *= 0.033. The Bedtime Resistance score of children taking more than 2 types of ASMs was 8.87 ± 2.87, which was higher than that of children taking only one drug, *p *= 0.023. The Parasomnias score of children with seizure control over 3 years was 6.65 ± 1.10, which was significantly lower than that of other groups, *p *= 0.045.

The anxiety self-rating scale of caregivers of children with uncontrolled seizures was 33.87 ± 9.92, which was also significantly higher than that of children with seizures controlled for more than 3 years, *p *= 0.014. When the number of caregivers was 1, the anxiety self-rating value of the caregivers was 34.93 ± 7.67, which was significantly higher than that when the number of caregivers was more than 2 (30.74 ± 8.00), *p *= 0.001. The caregiver anxiety self-rating scale score of children with the longest seizure duration of more than 5 min was 37.69 ± 9.00, which was significantly higher than that of children with the longest seizure duration of less than 5 min, *p *< 0.05.

There was no statistically significant difference between groups in the effect of COVID-19 infection on the sleep quality of CWEs and the anxiety of their caregivers.

## Discussion

Our study found that 10 children (4.2%) had seizures that reappeared within 1 month after the COVID-19 infection 4 cases (3.6%) had increased times of seizures, and 4 children (3.6%) had worsened seizures. Previous studies have reported that the proportion of patients ranged from 8%–35% with increased seizures during the COVID-19 epidemic, regardless of whether they were infected with COVID-19 (7–11), and the majority of those surveys during the pandemic showed that seizure frequency did not change, and seizure exacerbations occurred in less than 10 percent of patients, and these exacerbations may also be the result of natural fluctuations in epilepsy (12–15). The above studies suggest that COVID-19 infection does not affect seizures in patients with epilepsy. This result was surprising because fever and systemic infection are well-known triggers of seizures. In addition, in the long-term follow-up after the COVID-19 infection, it was found that half of the patients whose epilepsy control deteriorated during the COVID-19 infection recovered to the baseline level during the recovery period, and only 4.6% of the patients had increased seizure frequency after infection, while the vast majority of patients with stable epilepsy control after infection.

The average sleep time of 312 children with epilepsy in this study was 9.25 ± 1.04 h. The total score of the CSHQ was 37.25 ± 5.19 points, and there were 44 children (14.1%) with sleep quality problems. The children with earlier onset time, seizure duration longer than 5 min, seizures not well controlled, and taking more than 2 antiepileptic drugs had worse performance on the total score of the CSHQ. Epilepsy and sleep have a close, complex and reciprocal relationship. Sleep disturbances are approximately twice as common in people with epilepsy as in healthy controls, with approximately one-third of people with epilepsy reporting sleep disturbances and as many as half of children with newly diagnosed epilepsy having sleep disturbances at diagnosis (3, 16,17). Previous studies have shown that children with uncontrolled epilepsy have significantly higher CSHQ scores in terms of abnormal sleep and sleep-disordered breathing than children with controlled epilepsy, indicating that seizures can lead to abnormal sleep (18), which is basically consistent with our study. Since most of the cases in this study were well controlled, the number of children with sleep disorders in our cases was relatively small, which was lower than that reported in the literature.

This study shows that 2019-nCoV infection has no significant effect on the sleep quality of children. In terms of sleep time, children with 2019-nCoV infection were slightly shorter than children without infection, which may be due to infection symptoms such as fever, cough, and muscle pain. Several studies showed that (14, 19–26), epilepsy patients experienced sleep disturbances during COVID-19. Six of these cross-sectional studies reported a prevalence of sleep disturbance and insomnia of 7.1%–71.2% and 28.2%, respectively. Giordano et al. (19) measured sleep disturbances with the Insomnia Severity Inventory (ISI). Their study reported that the rate of sleep disturbance was 7%. However, the study by Assenza et al. (22) showed no significant difference in sleep quality between epileptic patients and healthy individuals.

This study suggests that caregivers of CWEs whose longest duration of seizures lasted more than 5 min, poor seizure control, and single caregivers were more likely to have anxiety. Previous studies have shown that the proportion of parents of children with epilepsy who have symptoms of depression and anxiety is 23.51%, which is higher than the proportion of parents of children without epilepsy who have symptoms of depression and anxiety (10.84%) (27). A study by Sanchez-Larsen et al. (28) suggested that increased caregiving stress or anxiety was associated with higher seizure frequency and worsening seizures. This study also showed that prolonged seizure duration and poor seizure control were the main causes of caregiver anxiety. In addition, when only one person takes care of children with epilepsy, the caregiver's anxiety self-score value was 34.93 ± 7.67, which is significantly higher than that of caregivers who share the burden of caregiving. This finding is consistent with previous studies (29). Fortunately, the COVID-19 infection did not aggravate the anxiety of the caregivers. The reason may be related to the scientific and rational understanding of the COVID-19 infection by the public, as well as the complete medical system and convenient medical care after infection.

This study has the following limitations. First, it only collected the data from one hospital, and the sample size was relatively small, which may not reflect the situation of a certain region or even the whole country. Second, the follow-up time was short, and there was no further study on the impact of secondary infection and the long-COVID on children's seizures and caregivers’ anxiety. Third, there is no control group, which cannot reflect the changes in sleep quality and anxiety caused by the COVID-19 infection in children with epilepsy and their caregivers compared with healthy people.

## Conclusions

The COVID-19 infection did not cause an increase in seizures in CWEs, nor did it worsen sleep quality in them, nor increase anxiety in their caregivers.

## Data Availability

The raw data supporting the conclusions of this article will be made available by the authors, without undue reservation.
